# RETRACTION: A Survey and Proposed Framework on the Soft Biometrics Technique for Human Identification in Intelligent Video Surveillance System

**DOI:** 10.1155/bmri/9850371

**Published:** 2025-08-08

**Authors:** BioMed Research International

RETRACTION: M-G. Kim, H-M. Moon, Y. Chung, and S.B. Pan, “A Survey and Proposed Framework on the Soft Biometrics Technique for Human Identification in Intelligent Video Surveillance System,” *BioMed Research International* 2012 (2012): 614146, https://doi.org/10.1155/2012/614146.

The above article, published online on 16 July 2012 in Wiley Online Library (http://wileyonlinelibrary.com), has been retracted following the agreement of the journal's Section Editor, Letterio S. Politi; and John Wiley & Sons Ltd.

The retraction has been agreed following an investigation by the publisher, which identified substantial overlap with an article published earlier by the same authors [1].

The authors were informed of this retraction but did not provide a response.
